# Surgical Treatment of Gastrointestinal Cancers

**DOI:** 10.3390/cancers15143743

**Published:** 2023-07-24

**Authors:** Ulrich Ronellenfitsch

**Affiliations:** Department of Visceral, Vascular and Endocrine Surgery, University Medical Center Halle (Saale), Martin-Luther-University Halle-Wittenberg, Ernst-Grube-Str. 40, 06120 Halle (Saale), Germany; ulrich.ronellenfitsch@uk-halle.de; Tel.: +49-345-557-2327

Even though there have been remarkable advances in systemic treatment of gastrointestinal malignancies over the last few decades, in the vast majority of instances, surgery remains the sole therapeutic approach offering a chance for a definite cure. Moreover, non-curative operations are part of the treatment algorithm of many patients with gastrointestinal cancer with the aim of alleviating symptoms, improving quality of life, and, in specific settings, prolonging survival [1]. In order to achieve the best outcomes, objective and unbiased decisions on if and when an operation should be carried out or rather avoided or deferred have to be made. Ideally, such decisions rely on a sound evidence base and are reached in a multidisciplinary setting [2]. Once the decision to operate has been made, it is on the surgeon to choose the most appropriate surgical approach for the specific patient and his or her disease from an ever-growing choice of available techniques. Minimally invasive operations, employing laparoscopy and thoracoscopy either in a conventional or robotic manner, have long gained popularity among both patients and surgeons. Yet, a broad foundation of high-quality evidence showing clearly superior outcomes for minimally invasive access does still not exist for many procedures [3].

Besides the indication for or against surgery and the choice of surgical techniques, perioperative treatment plays a crucial role in achieving the desired outcomes. This holds true for oncological surgery and particularly so for surgical treatment of gastrointestinal cancers. The perioperative treatment, possibly comprising neoadjuvant chemotherapy or radiotherapy but also measures to prepare patients for surgery and to ensure good postoperative outcomes, needs to be tailored to the characteristics of both the operation and the patient [4,5]. Postoperative complications, which can have a debilitating or even fatal outcome in extensive oncological resections, are dreaded by patients and surgeons alike. Common sense dictates that all efforts should be made to avoid complications, or, if this is not possible, detect them early in order to treat them in a timely and appropriate manner, in order to decrease the likelihood of failure to rescue [6]. Long-lasting or permanent sequelae for the patient need to be avoided or at least mitigated.

In this Special Issue of *Cancers*, the articles deal with the indication for and timing of surgery in specific scenarios. Gorbudhun et al. analyzed a cohort of patients with borderline-resectable pancreatic ductal adenocarcinoma who underwent neoadjuvant FOLFIRINOX or gemcitabine-based chemotherapy prior to resection [7]. The surgical and oncological results of their series are remarkable with a 75% complete resection rate and a median overall survival of 29 months. These data complement the recently published results of a randomized controlled trial suggesting that neoadjuvant chemotherapy can increase overall survival in patients with pancreatic cancer [8]. Folkestad et al. analyzed patients with duodenal neuroendocrine tumors and showed that only 1 of 23 patients who underwent endoscopic or surgical resection experienced recurrence [9]. They concluded that tumors < 10 mm can be treated endoscopically whereas larger ones should be treated surgically. Analyzing series of patients with such rare tumors is of particular importance given that randomized trials are hardly feasible in these entities. Taliadoros et al. focused on another rare gastrointestinal cancer, anal canal carcinoma, and performed a systematic review of the available evidence [10]. Based on their review, trimodality treatment with neoadjuvant chemoradiotherapy followed by abdominoperineal rectum resection seems to be the most effective approach. The last contribution dealing with surgical indications looked at para-aortic lymph node metastases from colorectal cancer. Zizzo et al. performed a systematic review but found no sufficient evidence to provide a recommendation for or against surgery in this situation [11].

Three articles of this Special Issue focused on specific surgical approaches and techniques. Kremer et al., based on a series of 16 patients with tracheoesophageal fistula, devised an algorithm for the treatment of this complex and multi-faceted entity, which is a severe manifestation of head and neck and esophageal cancers [12]. In their series, Pfitzmaier et al. evaluated a 1318 nm diode laser in liver surgery and showed convincing intra- and postoperative results of this device which hitherto had been predominantly employed in pulmonary and urological resections [13]. Lastly, Bizzoca and colleagues compared early postoperative results of laparoscopic and open resection in a propensity-matched sample of obese patients (BMI ≥ 30 kg/m^2^) with colorectal cancer [14]. They found a higher lymph node yield, faster recovery, and fewer complications in patients operated laparoscopically. These results provide an instructive addition to those of randomized trials comprising patients regardless of obesity, showing short-term advantages of minimally invasive procedures [15]. In a similar approach using propensity-score matching, Koo et al. showed similar short- and long-term outcomes for laparoscopic and open total gastrectomy in patients with mostly early-stage stomach cancer [16]. These findings are in line with higher-level evidence from randomized trials [17].

Petrou et al. contributed two manuscripts to the Special Issue, which are based on a review of guidelines and address perioperative issues. In the first work, they recommend the use of oral antibiotic prophylaxis, with or without mechanical bowel preparation, as an adjunct to intravenous antibiotic prophylaxis, in patients undergoing elective colorectal resections [18]. However, the evidence from randomized controlled trials is heterogeneous, and ongoing network meta-analyses might be able to provide clearer recommendations [19]. For obese patients with colorectal cancer undergoing surgery, they recommend key perioperative treatment steps comprising preoperative optimization by dieticians and physiotherapists, postoperative early mobilization, incentive spirometry, and general and chest physiotherapy as well as weight-adjusted doses of postoperative pharmacotherapy [20].

In summary, the contributions to this Special Issue provide important evidence on the many facets of surgical treatments for gastrointestinal cancers. By producing such evidence and translating it into clinical practice, the treatment outcomes of patients can be sustainably improved.
